# Supplementary material for: Sporothrix brasiliensis and Feline Sporotrichosis in the Metropolitan Region of Rio de Janeiro, Brazil (1998–2018)
Source: J Fungi (Basel). 2022 Jul 20;8(7):749. doi: 10.3390/jof8070749 (PMC9325134; doi:10.3390/jof8070749)
Supplement: Supplementary file 1 [file jof-08-00749-s001.zip › jof-1728197-supplementary.pdf]

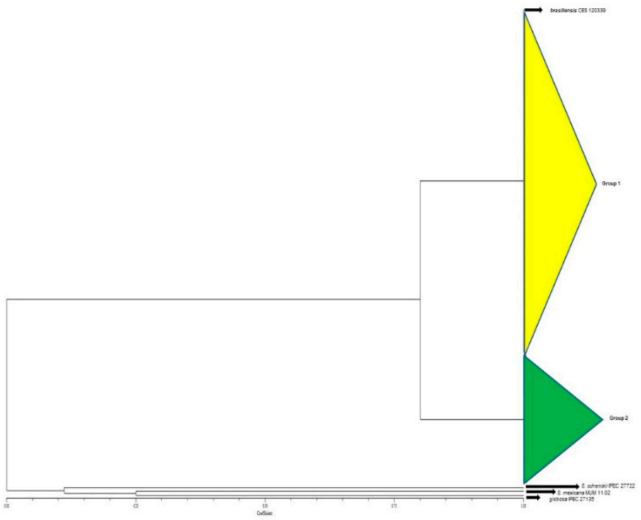

Figure S1: Phylogenetic tree showing species identification and the degree of similarity between T3B fingerprinting profiles (Group 1 without intraspecific variation and Group 2 with intraspecific variation) among the *Sporothrix* isolates obtained of the 119 cats at the Laboratory of Clinical Research on Dermatozoonoses in Domestic Animals (INI)/Fiocruz, Rio de Janeiro, Brazil, 1998 to 2018 using the UPGMA cluster method.

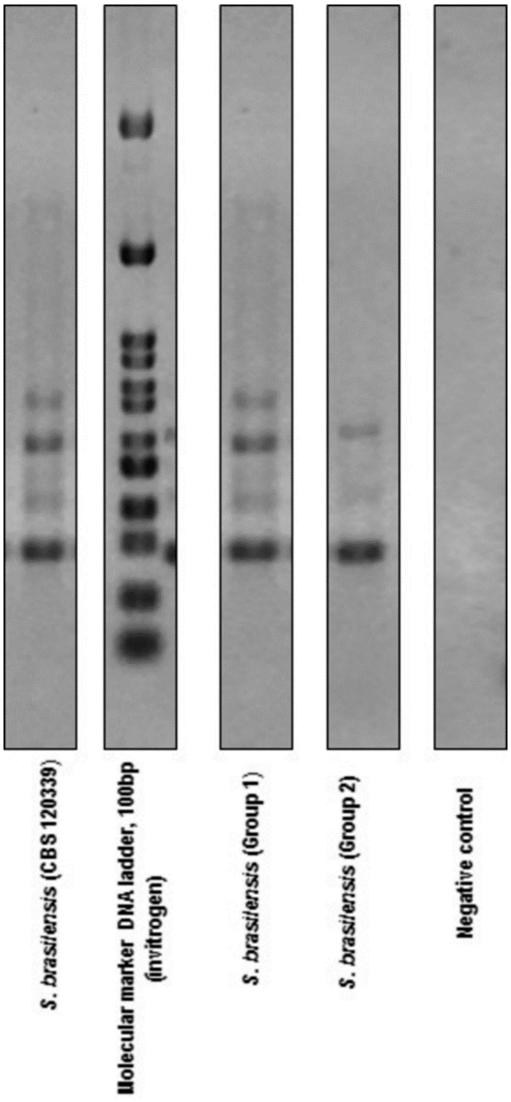

Figure S2: Representative PCR fingerprinting profiles obtained with primer T3B for *Sporothrix basiliensis* isolates. Lanes 1 and 5: (1) *S. brasiliensis* CBS 120339 (2) Molecular marker DNA ladder 100 bp; (3) *S. brasiliensis* (Group 1 - without intraspecific variation); (4) *S. brasiliensis* (Group 2 - with intraspecific variation); (5) Negative control.
